# Development of 3D-Printed Collagen Scaffolds with In-Situ Synthesis of Silver Nanoparticles

**DOI:** 10.3390/antibiotics12010016

**Published:** 2022-12-22

**Authors:** Sofia Municoy, Pablo Edmundo Antezana, Martín Gonzalo Bellino, Martín Federico Desimone

**Affiliations:** 1 Universidad de Buenos Aires, Facultad de Farmacia y Bioquímica, Instituto de Química y Metabolismo del Fármaco (IQUIMEFA), Consejo Nacional de Investigaciones Científicas y Técnicas (CONICET), Junín 956, Buenos Aires 1113, Argentina; 2 Instituto de Nanociencia y Nanotecnología, Comisión Nacional de Energía Atómica, Consejo Nacional de Investigaciones Científicas y Técnicas (CONICET), San Martín 1650, Argentina

**Keywords:** 3D-printing, UV irradiation method, AgNPs, collagen, antimicrobial scaffolds, post-printing

## Abstract

UV-irradiation method has grown as an alternative approach to in situ synthetize silver nanoparticles (AgNPs) for avoiding the use of toxic reducing agents. In this work, an antimicrobial material by in situ synthesizing AgNPs within 3D-printed collagen-based scaffolds (Col-Ag) was developed. By modifying the concentration of AgNO_3_ (0.05 and 0.1 M) and UV irradiation time (2 h, 4 h, and 6 h), the morphology and size of the in situ prepared AgNPs could be controlled. As a result, star-like silver particles of around 23 ± 4 μm and spherical AgNPs of 220 ± 42 nm were obtained for Ag 0.05 M, while for Ag 0.1 M cubic particles from 0.3 to 1.0 μm and round silver precipitates of 3.0 ± 0.4 μm were formed in the surface of the scaffolds at different UV irradiation times. However, inside the material AgNPs of 10–28 nm were obtained. The DSC thermal analysis showed that a higher concentration of Ag stabilizes the 3D-printed collagen-based scaffolds, while a longer UV irradiation interval produces a decrease in the denaturation temperature of collagen. The enzymatic degradation assay also revealed that the in situ formed AgNPs act as stabilizing and reinforcement agent which also improve the swelling capacity of collagen-based material. Finally, antimicrobial activity of Col-Ag was studied, showing high bactericidal efficiency against Gram-negative (*Escherichia coli*) and Gram-positive (*Staphylococcus aureus*) bacteria. These results showed that the UV irradiation method was really attractive to modulate the size and shape of in situ synthesized AgNPs to develop antimicrobial 3D-printed collagen scaffolds with different thermal, swelling and degradation properties.

## 1. Introduction

The recent advances in 3D printing technology and its marriage with the biomaterials field allowed the development of 3D-printed scaffolds for biomedical applications [1]. This technology leads to the manufacture of custom-made 3D structures with high resolution and controlled architecture [2]. Among the different methods used to create 3D-materials, fuse deposition modelling (FDM) is highly used, and it is based on the material extrusion [3]. In this sense, extrusion-based printing is an adaptation from the FDM with the purpose of printing biocompatible materials and cells. This method used pneumatic pressure or mechanical force to extrude the ink out of the nozzle in an uninterrupted line [4].

There are several materials that could be used to produce 3D-printed scaffolds, such as collagen, chitosan, cellulose, hyaluronic acid, and alginate. Among them, collagen has gained popularity because of its interesting properties. Collagen is the main protein of the extracellular matrix of animals, almost 30% in vertebrates. Maintaining the biological and structural integrity of the extracellular matrix is the main role of collagen [5]. Collagen type I has important biocompatible properties and forms strong and stable fibers through its self-assembly and cross-linking that could lead to the development of useful scaffolds [6,7]. On the other hand, it is interesting to improve the properties of the scaffolds by adding other functionalities to the 3D-printed material. In this sense, the incorporation of antimicrobial agents is a relevant improvement. In this line, Ag nanoparticles (AgNPs) has gained popularity as a metal with great antimicrobial activity [8,9]. This metal has an important antibacterial capacity against both Gram-negative and Gram-positive bacteria, even the drug-resistant ones [10,11]. AgNPs has several mechanisms to achieve the antimicrobial activity: anchoring and penetrating the bacterial cell wall, leading to structural changes in cell membrane; binding to proteins; interacting with DNA bases leading to irreversible damages; production of free radicals; and inhibition of cell division and reproduction [12,13,14]. The presence of these different mechanisms of action, hinders the development of microbial resistance. It is known that the main responsible of the AgNPs antimicrobial activity is the silver ions release (Ag^+^) [15].

There are several physical and chemical methods available to synthesize AgNPs, such as electrochemical changes, and chemical and photochemical reduction [16,17,18,19]. Among them, the chemical reduction is the most frequently used in order to reduce Ag^+^ ions to obtain AgNPs. In this sense, AgNPs are usually synthesized by reduction of a silver salt with a reducing agent, such as sodium borohydride in the presence of a colloidal stabilizer. The most commonly used colloidal stabilizers are polyvinyl alcohol [20], poly(vinylpyrrolidone) [21], bovine serum albumin [22], and citrate [23]. In another work, polyethyleneimine was used to avoid the aggregation of AgNPs prepared on the surface of commercial cellulose filter papers by reduction with polydopamine [24]. Supramolecular agents, such as cyclophanes, were also used to stabilized AgNPs [25]. During the process of synthesizing the nanoparticles the different parameters, such as the interaction kinetics of the metal ions with the reducing agent and adsorption of the stabilizer agent, among others, can influence the morphology, stability, and physicochemical properties of AgNPs [26,27]. However, the reduced agent used during these chemical methods, such as sodium borohydride, hydrazine, and N,N-dimethylformamide could lead to environmental concerns due to their inherent toxicity [28]. To overcome this disadvantages, it is gaining attention the use of physical methods, such as photoreduction methods, like ultrasonic waves, laser, and ultraviolet light (UV) irradiation [29,30,31,32]. In particular, UV light has become a promising method to in situ synthesize AgNPs from silver solutions without using any reducing or stabilizer agent [33]. This leads to a method which is ecofriendly, with increased energy efficiency and reduced costs [34].

Since it is promising the development of materials that could be printed with 3D technology, the aim of this work was to develop a new 3D-printed material based on collagen, with the in situ synthesis of AgNPs in order to endow the biomaterial with the antimicrobial activity of silver.

## 2. Results and Discussion

### 2.1. Preparation of Antimicrobial 3D-Printed Collagen-Based Scaffolds and In situ Reduction in Ag

The first step for the manufacture of the antimicrobial 3D-printed AgNPs-loaded Collagen gels (Col-Ag) involved the preparation of suitable inks. In order to obtain a collagen-based ink with good printability, a high concentration of collagen was needed to enhance the shape fidelity. For this reason, a collagen solution in acetic acid was lyophilized and then resuspended in distilled water or AgNO_3_ solutions to obtain a final concentration of collagen of 80 mg/mL. After 24 h of hydration and mixing, homogeneous inks were obtained, and used to print the 3D scaffolds.

**Figure 1:**
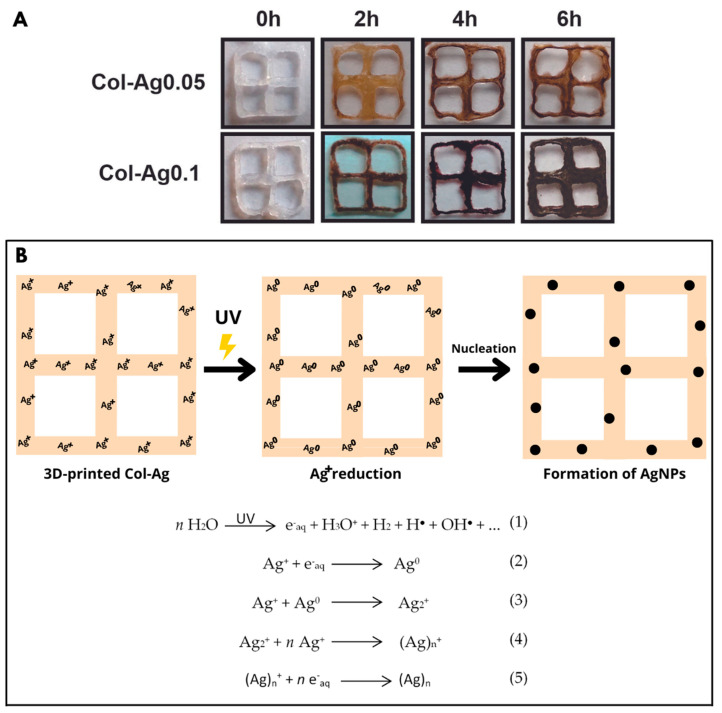
(**A**) Images of dry 3D-printed collagen-based scaffolds after in situ reduction of Ag by different UV irradiation intervals. Dimensions of 3D-printed collagen-based scaffolds are 6 mm × 6 mm × 2 mm. (**B**) Schematic illustration of the mechanism of in situ AgNPs formation by using the UV irradiation method.

After printing, 3D square grid collagen-based scaffolds (6 mm × 6 mm × 2 mm) were obtained as test samples. To induce in situ reduction in Ag, 3D-printed Col-Ag grids were exposed to UV light during different time intervals. Once jellified, 3D-printed collagen-based composites were finally obtained from the different AgNO_3_ 0.05 and 0.1 M solutions, labeled as Col-Ag0.05 and Col-Ag0.1, respectively. As can be seen in Figure 1A, colorless 3D-printed Col-Ag scaffolds were obtained before UV irradiation, but after 2 h, 4 h, and 6 h of exposure to UV light, a gradual increase in brown color was observed as a result of the reduction in silver and a possible formation of silver particles within the collagen network. In addition, the brown color was more intense in Col-Ag0.1 than in Col-Ag0.05, due to a greater concentration of silver in the ink.

**Figure 2:**
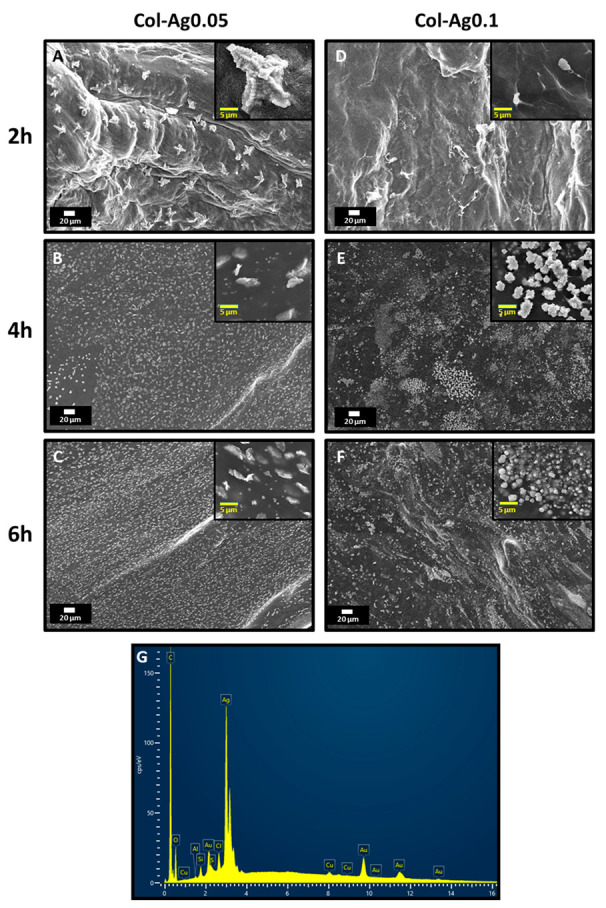
SEM images of (**A**): Col-Ag0.05-2; (**B**): Col-Ag0.05-4; (**C**): Col-Ag0.05-6; (**D**): Col-Ag0.1-2; (**E**): Col-Ag0.1-4; (**F**): Col-Ag0.1-6. (**G**): Representative EDS analysis of Col-Ag.

**Figure 3:**
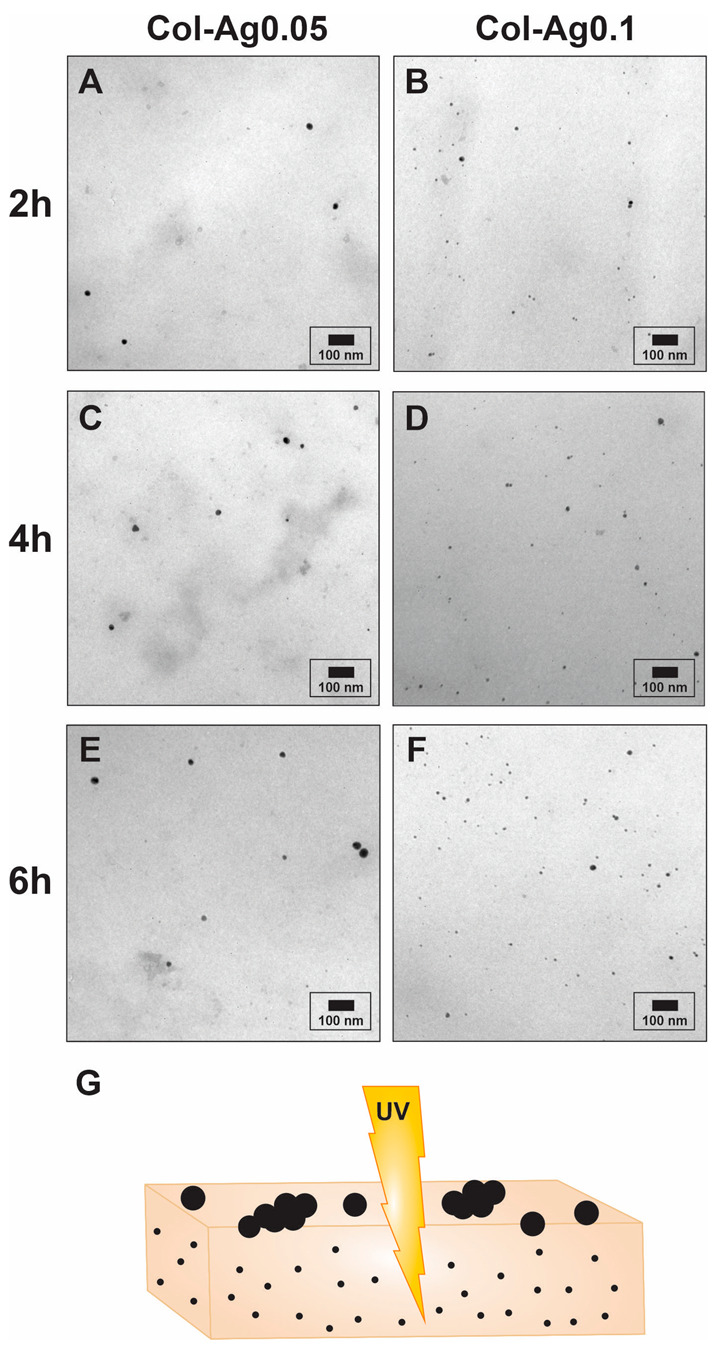
TEM images of the AgNPs formed inside the 3D-printed collagen-based scaffolds. (**A**): Col-Ag0.05-2; (**B**): Col-Ag0.05-4; (**C**): Col-Ag0.05-6; (**D**): Col-Ag0.1-2; (**E**): Col-Ag0.1-4; (**F**): Col-Ag0.1-6. (**G**): schematic illustration of the variation of the size of the silver particles according to the place of the material where they are formed.

**Figure 4:**
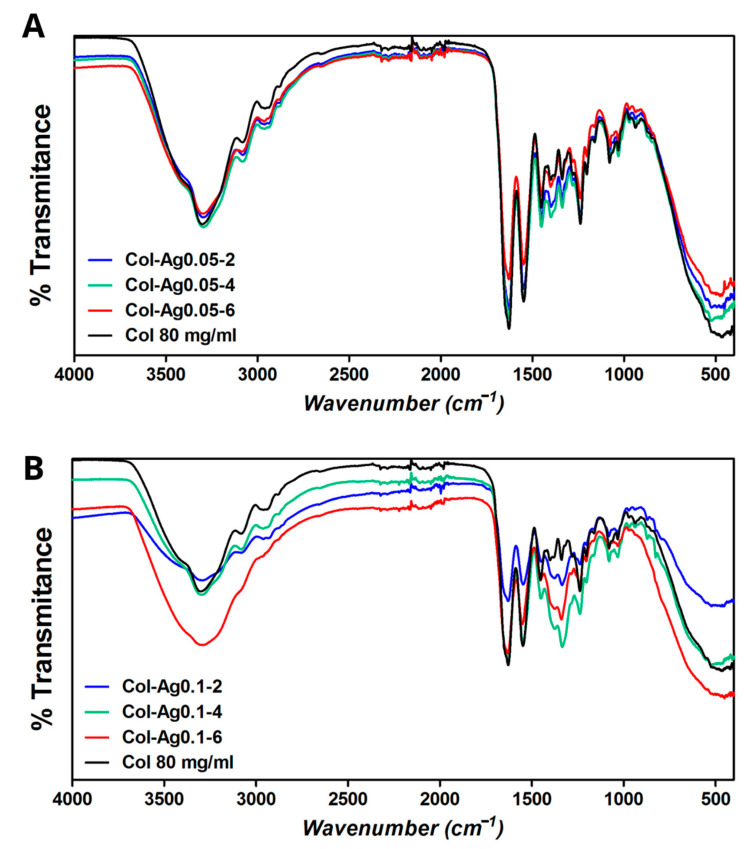
FTIR spectra of Col, Col-Ag0.05 (**A**), and Col-Ag0.1 (**B**) after different time of UV light exposure (0 h, 2 h, 4 h, and 6 h).

**Figure 5:**
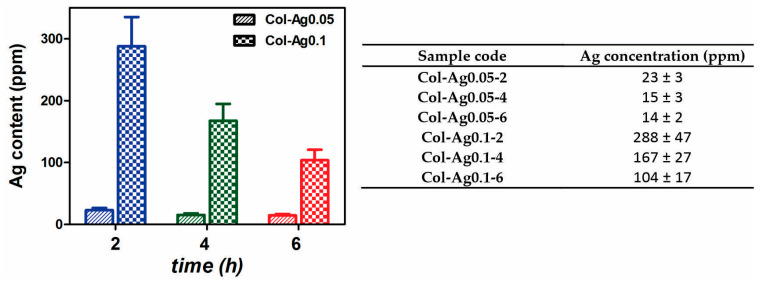
Silver content in 3D-printed Col-Ag0.05 and Col-Ag0.1 scaffolds, after 2 h, 4 h, and 6 h of UV irradiation, measured by Atomic Absorption spectroscopy.

The mechanism proposed by which silver is reduced within collagen suggests the formation of hydrated electrons (e^−^_aq_) by photolysis of aqueous solution during UV irradiation (Figure 1B-1) and the following capture of these electrons by Ag^+^ ions (Figure 1B-2). The neutral Ag^0^ atoms bind to other Ag^+^ ions to give Ag_n_^+^ species (Figure 1B-4), which in presence of the hydrated electrons leads to the formation of silver particles (Figure 1B-5) [32]. It was found that the high rate constant of the reaction between e^−^_aq_ and Ag^+^ in a solution of a biopolymer indicates that silver ions are favorably reduced to Ag^0^ by UV irradiation [35]. At the same time, the amino acids glycine, proline, and hydroxy proline present in collagen could be involved in the Ag^+^ ions reduction [36,37].

### 2.2. Characterization of Antimicrobial 3D-Printed Col-Ag

#### 2.2.1. SEM Imaging

To confirm the formation of silver particles within collagen hydrogels after UV irradiation, the 3D-printed materials were analyzed by SEM. Figure 2 shows SEM images obtained for 3D-printed named Col-Ag0.05-2, Col-Ag0.05-4, Col-Ag0.05-6, Col-Ag0.1-2, Col-Ag0.1-4, and Col-Ag0.1-6, where the second tags denote the UV irradiation time. Silver particles with different morphology and sizes were formed on the surface of the scaffolds depending on the initial concentration of silver used to prepare the inks and on the UV irradiation time. In case of Col-Ag0.05-2, star-like micro-sized particles [38] of around 23 ± 4 μm were obtained (Figure 2A), but as UV irradiation interval increased to 4 h and 6 h (Figure 2B,C, respectively), a mixture of free spherical AgNPs [20] of 220 ± 42 nm and elongated clusters of around 4.0 ± 0.6 μm formed by the agglomeration of the AgNPs were observed. Col-Ag0.1-2 (Figure 2D) shows few round silver precipitates of around 3.0 ± 0.4 μm, whereas after 4 h and 6 h of UV irradiation cubic silver particles [39,40] from 0.3 to 1.0 μm were mainly observed in 3D-printed Col-Ag0.1-4 and Col-Ag0.1-6 (Figure 2E,F, respectively). Furthermore, at a longer time of UV radiation, the formation of a larger number of particles was observed. The analysis of the obtained nanomaterials by energy-dispersive analysis further confirms the presence of the silver in nanoparticles (Figure 2G).

From these results, it is evident that varying the condition of synthesis (concentration of AgNO_3_ and UV irradiation time), the formation process of silver particles changes, leading to particles of different size and morphology [41]. The shape of the Ag particles obtained depends on the interaction between Ag^+^ ions with collagen and electrons formed during the UV exposure, and the reaction time and the crystallinity of the initial nucleus. These factors could be controlled by modifying the concentration of AgNO_3_ used for the preparation of the ink and the molar ratio between collagen and AgNO_3_. In this sense, it was possible to tune the shape and size of in situ synthesized particles by a more environmentally friendly and rapid method, avoiding the use of toxic chemicals.

To further characterize the in situ prepared AgNPs, the material was completely degraded by a collagenase solution and the obtained suspension was studied by TEM (Figure 3). Interestingly, the particles formed inside the collagen have a smaller size (Table 1) than those formed on the surface of the material (Figure 2), possibly due to a stabilizing action of the collagen [42] and due to the different degree of UV light penetration among with limited space for particles growth (Figure 3G). This is attractive since the size and morphology are not only controlled through the different conditions of synthesis studied, but they are also given according to the place of the material they are formed, whether on the surface or inside the 3D-printed scaffolds.

#### 2.2.2. FTIR Analyses

FTIR spectra of 3D-printed Col (in the absence of AgNPs), Col-Ag0.05 and Col-Ag0.1 scaffolds are shown in Figure 4. FTIR analysis was performed to study whether there was an interaction between collagen and in situ synthesized Ag particles. When comparing FTIR spectra of Col, Col-Ag0.05 (Figure 4A) and Col-Ag0.1 (Figure 4B) representative peaks were found. However, a close analysis of the spectra indicates that a more intense band at around 1340 and 1400 cm^−1^ was observed for 3D-printed Col-Ag0.05 and more clearly in Col-A0.1 scaffolds. These vibration signals, located at 1340 and 1400 cm^−1^, respectively, could indicate the possible interaction between silver particles and collagen (antiparallel-βsheet) [43]. Furthermore, a decrease in the intensity of the bands at 1635 and 1556 cm^−1^ corresponding to C=O of amide I and NH groups of amine II of collagen was observed for these scaffolds. This may also indicate that these groups are involved in the reduction and stabilization of silver particles in collagen [36,44].

#### 2.2.3. Silver Content

Silver content in the 3D-printed Col-Ag materials was quantified by Atomic Absorption technique. Figure 5 shows the final concentration of silver present in Col-Ag0.05 and Col-Ag0.1 after the different UV irradiation times.

As expected, Ag concentration of Col-Ag0.1 is higher than in Col-Ag0.05. The reduction in Ag concentration from the initial amount of AgNO_3_ used for the preparation of the inks is related with the successive washes of the materials after the gelation with NH_3_. Interestingly, a longer exposure to UV radiation light produces a decrease in the final silver content in the 3D-printed collagen materials. This could be attributed to the higher amount of Ag particles in situ formed at longer exposure time to UV light (Figure 2). It has been probed that interaction between proteins and silver ions is stronger than with Ag^0^ formulations [45], thus, after successive washes, a higher percentage of Ag particles are prone to be released, remaining less Ag in contact with the collagen.

#### 2.2.4. DSC Thermal Analysis

**Figure 6:**
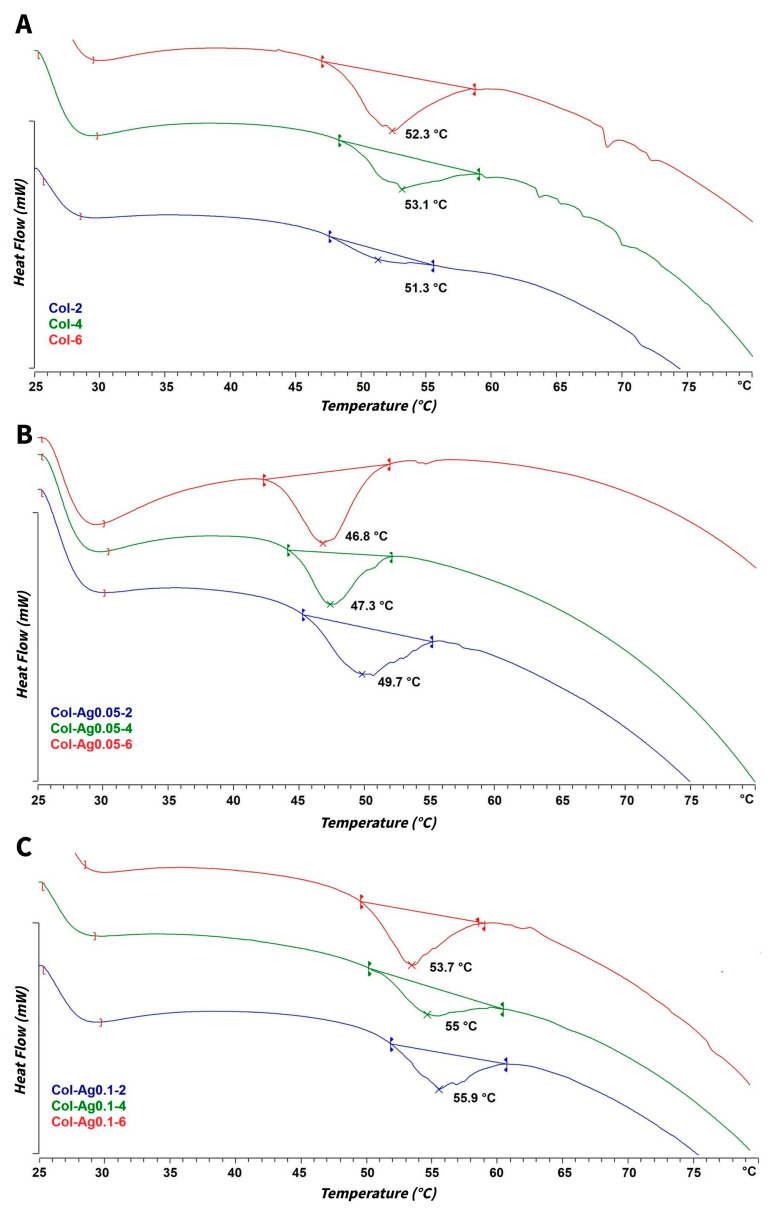
DSC thermal analyses for 3D-printed Col (**A**), Col-Ag0.05 (**B**), and Col-Ag0.1 (**C**) scaffolds after different UV irradiation times.

To determine the changes in the morphological integrity and stability of the 3D-printed collagen scaffolds after in situ synthesized Ag particles, the denaturation temperature of the collagen-based scaffolds was analyzed using DSC. Figure 6 shows the DSC curves for Col, Col-Ag0.05, and Col-Ag0.1 exposed to 2 h, 4 h, and 6 h of UV light.

DSC curves of 3D-printed Col scaffolds (Figure 6A) exposed at different UV times show the typical denaturation temperature at ca. 52.0 ± 0.9 °C found in fibrillar collagen [46]. Interestingly, for Col-Ag0.05 and Col-Ag0.1, opposite effects were found. In case of Col-Ag0.05 (Figure 6B), a shifting of DSC curves to lower temperatures is observed. This indicates that the addition of 0.05 M concentration of Ag produces a destabilization of collagen fibrils by interfering in the self-assembling processes of collagen molecules. This effect can be attributed to the binding of silver ions with collagen, mainly sulfhydryl groups of the fibrillar protein, which finally leads to a decrease in the denaturation temperature of the collagen [47,48]. However, when Col-Ag0.1 (Figure 6C) was analyzed, DSC curves were obtained at higher temperatures than Col. This suggests that under this conditions collagen is stabilized by a higher concentration of Ag (0.1 M) through other chemical interactions [49]. In fact, FTIR spectra indicate that the interactions between Ag and amides groups of collagen are stronger in Col-Ag0.1 than in Col-Ag0.05. This effect is in good agreement with previous data showing that silicic acid influenced the self-assembly of collagen triple helices and, consequently, their denaturation temperature. On the one hand, at low silicification rates, a slightly diminution in the denaturation temperature was attributed to the charges provided by the inorganic component which hinders the correct aggregation of collagen. On the other hand, a significant increase in collagen thermal stability was reported in the presence of higher silicate content due to the coverage of collagen fibril with the inorganic component that stabilized its structure [6,50].

Furthermore, the longer the UV irradiation time, the lower the denaturation temperature of collagen. This behavior was observed for both Col-Ag0.05 and Col-Ag0.1, demonstrating that an increase in the irradiation dose damages collagen molecules and produces conformational changes, such as a loss of triple helical content of the protein [51]. In addition, longer irradiation times lead to the formation of more particles and, consequently, less Ag^+^ ions are available to interact with collagen. From these results, it could be assumed that stability of collagen matrices depends on Ag concentration in the ink and the UV radiation dose.

#### 2.2.5. Enzymatic Degradation

**Figure 7:**
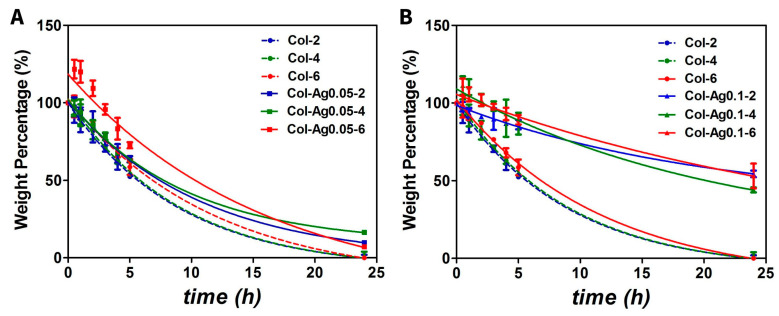
Collagenase degradation test of 3D-printed collagen-based scaffolds. Weight percentage is shown as a function of time comparing Col with Col-Ag0.05 (**A**), and Col with Col-0.1 (**B**). Col-2(--●--blue), Col-4(--●--green), Col-6 (--●--red), Col-Ag0.05-2 (-■-blue), Col-Ag0.05-4 (-■-green), Col-Ag0.05-6 (-■-red), Col-Ag0.05-2 (-▲-blue), Col-Ag0.05-4 (-▲-green), and Col-Ag0.05-6 (-▲-red). Results are expressed as mean ± SD from triplicate experiments.

Stability of the 3D-printed collagen-based hydrogels was also evaluated by collagenase degradation test. For this, Col, Col-Ag0.05 and Col-Ag0.1 were incubated with a solution of collagenase 20 U/mL at 37 °C and their weight was measured over time. As a result, after 24 h Col without Ag was completely degraded and Col-Ag0.05 preserved around 10% of its initial weight (Figure 7A). In contrast, 3D-printed Col-Ag0.1 scaffolds only lost about 50% of their initial mass (Figure 7B) indicating, and the thermal analysis, that the in situ formation of silver particles from a higher Ag concentration improves stabilization of 3D-printed fibrillar collagen. According to previous works, silver particles act as reinforcement of collagen-based material enhancing its stability and slowing down collagenase degradation by a physical filling effect and chemical interactions [20,52].

#### 2.2.6. Swelling Capacity

**Figure 8:**
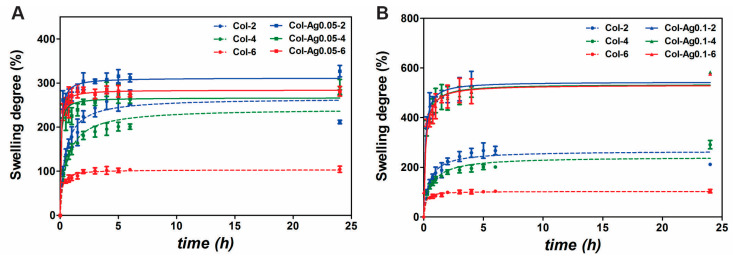
Swelling capacity of Col-Ag0.05 (**A**) and Col-Ag0.1 (**B**). Col-2(--●--blue), Col-4(--●--green), Col-6 (--●--red), Col-Ag0.05-2 (-■-blue), Col-Ag0.05-4 (-■-green), Col-Ag0.05-6 (-■-red), Col-Ag0.05-2 (-▲-blue), Col-Ag0.05-4 (-▲-green), and Col-Ag0.05-6 (-▲-red). Results are expressed as mean ± SD from triplicate experiments.

The swelling response of the 3D-printed Col-Ag materials was also investigated as it is related to the exchange of substances, such as Ag ions and Ag particles, and subsequently, to their antimicrobial activity [53]. Figure 8 shows that Col, Col-Ag0.05, and Col-Ag0.1 reach the maximum water uptake after 6 h, and no further change was observed after 24 h. Col-Ag0.01 (Figure 8B) exhibited a higher capacity to take up water compared to Col-Ag0.05 (Figure 8A), and both showed a higher water absorbency than Col. As the number of fixed charges in collagen increases in presence of a higher Ag concentration, the electrostatic repulsions between collagen chains also increases. As a result, hydrophilicity of the collagen network is increased, leading to a greater swelling capacity [54]. Furthermore, it was observed that at longer UV irradiation intervals the swelling capacity decreases, more significantly in 3D-printed Col scaffolds. According to DSC results, UV radiation causes a possible collapse of the structure and damage of collagen [55,56].

### 2.3. Antimicrobial Activity of 3D-printed Col-Ag Scaffolds

**Figure 9:**
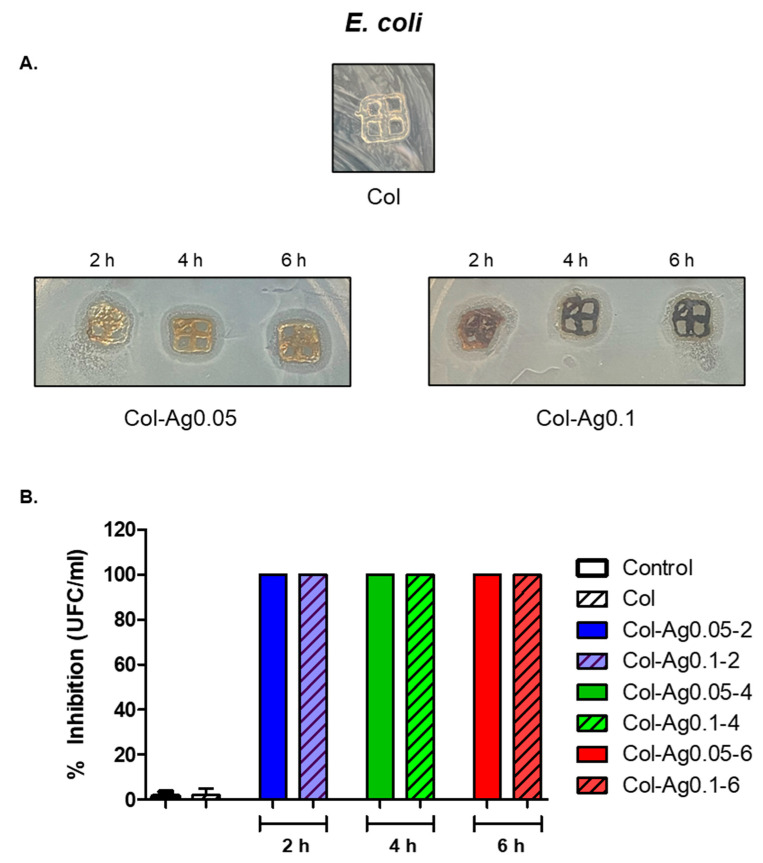
Antimicrobial activity of 3D-printed Col and Col-Ag gels against *E. coli*. (**A**) Disk diffusion method. (**B**) Percentage of growth inhibition, evaluated by the dilution method.

Antimicrobial activity of 3D-printed Col-Ag was studied against two bacterial strains, *E. coli* and *S. aureus*, using two different methods, the solid and the dilution technique. As it is shown in Figure 9A, when the antimicrobial activity was evaluated against *E. coli*, there were clear inhibition zones both around the samples and between the gaps for Col-Ag0.05-2, Col-Ag0.05-4, Col-Ag0.05-6, Col-Ag0.1-2, Col-Ag0.1-4, and Col-Ag0.1-6. When inhibition zones with commercial nanoparticles were studied, it was observed that the inhibition zone for the Ag concentration of 288 ppm was (1.3 ± 0.05 cm) similar to the inhibition zone obtained for Col-Ag0.05-6 (1.0 ± 0.06 cm). This result shows that collagen material with Ag presented a higher antimicrobial activity compared to Ag alone. This may be due to the fact that collagen’s own hydration improves the silver diffusion phenomenon [57]. In addition, when the antimicrobial activity was evaluated by the dilution method, 100% inhibition of bacterial growth was observed for Col-Ag0.05-2, Col-Ag0.05-4, Col-Ag0.05-6, Col-Ag0.1-2, Col-Ag0.1-4, and Col-Ag0.1-6 (Figure 9B). These results clearly demonstrate that 3D-printed Col-Ag scaffolds present antimicrobial activity against Gram-negative bacteria. In addition, the MIC for Gram-negative bacteria was 4.66 ppm.

**Figure 10:**
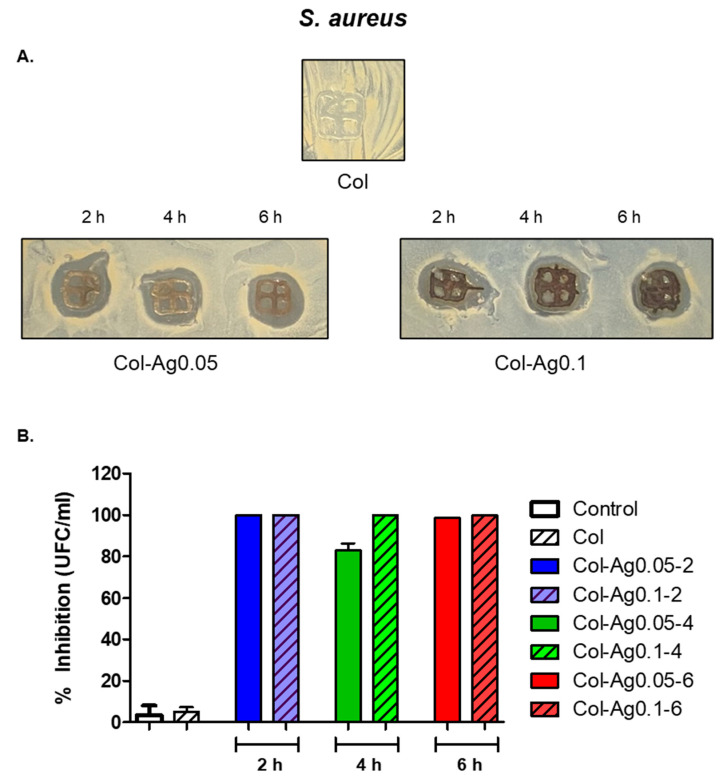
Antimicrobial activity of silver in 3D-printed Col and Col-Ag gels against *S. aureus*. (**A**) Disk diffusion method. (**B**) Percentage of growth inhibition, evaluated by the dilution method.

When the antimicrobial activity against *S. aureus* was studied by the solid method, well-defined inhibition zones were also observed around the samples and between the gaps for Col-Ag0.05-2, Col-Ag0.05-4, Col-Ag0.05-6, Col-Ag0.1-2, Col-Ag0.1-4, and Col-Ag0.1-6 (Figure 10A). When inhibition zones with commercial nanoparticles was studied, it was observed that the inhibition zone for the Ag concentration of 167 ppm was (1.2 ± 0.07 cm) equal to the inhibition zone obtained for Col-Ag0.05-6 (1.2 ± 0.05 cm). This result shows that collagen material with Ag presented a higher antimicrobial activity compared to Ag alone. This may be due to the fact that collagen’s own hydration improves the silver diffusion phenomenon. Moreover, as it is shown in Figure 10B, when evaluated the antimicrobial activity using the dilution method, it was found a percentage of inhibition of bacterial growth greater than 90% for Col-Ag0.05-2, Col-Ag0.05-6, Col-Ag0.1-2, Col-Ag0.1-4, and Col-Ag0.1-6, except for Col-Ag0.05-4. These results suggest that 3D-printed Col-Ag materials also present antimicrobial activity against Gram-positive bacteria. In addition, the MIC for Gram-negative bacteria was 5.83 ppm. However, the slight difference between the percentage of inhibition of bacterial growth among both bacteria strains could be explained by the structural difference in the bacterial cell walls between Gram-negative and Gram-positive bacteria [58]. The antimicrobial activity observed against both bacterial strains is attributed to the reported well-known antibacterial capacity of the AgNPs thanks to the release of Ag^+^ ions [20,52]. In fact, when the materials were incubated for 24 h in liquid medium, it was found that Col-Ag0.05-2, Col-Ag0.05-4, Col-Ag0.05-6, Col-Ag0.1-2, Col-Ag0.1-4, and Col-Ag0.1-6 released 32.09 ± 0.37, 29.47 ± 0.38, 27.30 ± 1.86, 55.21 ± 0.40, 55.42 ± 0.31, and 56.44 ± 0.37 ppb of Ag^+^, respectively.

## 3. Materials and Methods

### 3.1. Preparation of Inks

Collagen (type I) was obtained from rat tails tendons and dissolved in 17.5 mM acetic acid. The collagen solution was lyophilized, then the dry powder was resuspended with the necessary volume of distilled water or a solution of AgNO_3_ in order to obtain a final collagen concentration of 80 mg/mL. Based on previous works, a concentration of 0.05 M [20,52] of AgNO_3_ was used to in situ prepared AgNPs, and for comparison a higher concentration 0.1 M was also evaluated. The three prepared inks were allowed to hydrate at 4 °C in darkness, and, after 24 h, were transferred to a 1 mL syringe.

### 3.2. Printing of Collagen-Based Scaffolds

A square grid design was chosen as model structure to study 3D-printed Col-Ag material properties. The design was created with the software Onshape^®^ (2013–Present) and saved as stereolithography (.stl) file, which was then converted to a G-code file by Repetier software. This G-code file was then used to dictate the scaffold dimensions and printing coordinates to the bioprinter. Scaffolds were printed using an extrusion-based Life SI 3D-bioprinter (Life SI, Humanizing Technology, Córdoba, Argentina). For this, the optimal printing conditions were first determined (25 °C temperature, delay of 40 μseg, and 1 nL/4 μm for the amount of printed material), then 6 mm × 6 mm × 2 mm square grids were printed by extruding the inks through a 21G needle with an internal diameter of 514 μm.

### 3.3. In situ Reduction in Ag by Using the UV Irradiation Method

To induce in situ reduction in silver nitrate, the UV irradiation method was used. The 3D scaffolds printed from the inks prepared with collagen dissolved in 0.05 or 0.1 M of AgNO_3_ were irradiated utilizing an ultraviolet light (UV) chamber with a UV lamp at λ: 365 nm. UV light exposure was applied during 2 h, 4 h, or 6 h at room temperature. As control, 3D-printed scaffolds obtained from collagen inks without AgNO_3_ were also irradiated with UV light.

### 3.4. Gelation of 3D-Printed Scaffolds

After UV irradiation, the in situ Ag particles-synthesized 3D-printed Col scaffolds were exposed to ammonia vapors to produce gelation of collagen. After 12 h, the 3D-printed gels were removed from the basic atmosphere, and finally washed with water until a neutral pH was reached. The hydrogels were defined as Col-2, Col-4, Col-6, Col-Ag0.05-2, Col-Ag0.05-4, Col-Ag0.05-6, Col-Ag0.1-2, Col-Ag0.1-4, and Col-Ag0.1-6, respectively, according to the silver concentration (0.05 or 0.1 M) and UV irradiation time (2 h, 4 h, and 6 h).

### 3.5. Characterization of 3D-Printed Col-Ag Scaffolds

#### 3.5.1. SEM Imaging

Col-Ag gels were characterized by scanning electron microscopy (SEM) using a Carl Zeiss-EVO 10 HV W microscope. For this, the 3D-printed Col-Ag gels were fixed with a 2.5% glutaraldehyde solution and lyophilized for 48 h. The freeze-dried samples were placed on carbon double-sided tape supported in aluminum SEM sample holder and sputter-coated with gold.

#### 3.5.2. TEM Imaging

The morphology and size of the in situ formed AgNPs were studied by transmission electron microscopy (TEM) using a Zeiss EM109T electron microscope. For this, the 3D-printed Col-Ag scaffolds were completely degraded by incubation with type I collagenase solution of 100 U/mL (Gibco^®^ (Waltham, MA, USA), 340 U/mg) at 37 °C for 12 h. Finally, a drop of each obtained suspension was added to carbon copper grids and let dry for a few minutes before introducing them to the microscope.

#### 3.5.3. FTIR Analysis

Fourier transform infrared (FTIR) spectra of 3D-printed collagen gels (Col and Col-Ag) were performed using an FTIR-Raman Nicolet iS50 (Thermo Scientific, Waltham, MA, USA). Briefly, the three-dimensional samples were dried under a nitrogen flow, then a small slice of each one was placed on the attenuated total reflection accessory of the spectrometer. The spectra were recorded over the range of 4000–500 cm^−1^. The SpectraGryph software (v.1.2.16) was used to normalize FTIR data.

#### 3.5.4. DSC

Differential Scanning Calorimetry (DSC) analysis of 3D-printed collagen gels (Col and Col-Ag) were performed using a DSC Calorimeter (DSC 822, Mettler Toledo, Switzerland). For this purpose, a known amount (5–10 mg) of each sample was placed in an aluminum crucible and the DSC curves were obtained with a heat flow of 10 °C min^−1^ from 25 to 80 °C, under a nitrogen atmosphere. The collagen denaturation temperature was determined at the maximum of the melting peak after baseline subtraction.

#### 3.5.5. Swelling Capacity

To determine the amount of liquid that 3D-printed collagen-based gels can absorb, Col and Col-Ag gels were dried and weighed. Then, they were re-hydrated with 500 μL of distilled water and incubated at room temperature. The change of the weight was measured over time for 24 h. For each time, the 3D-printed hydrogels were removed and wiped dry. In order to quantify the swelling capacity of each sample, the following Equation (1) was used:

(1) $$\%\;\mathrm{Swelling}\;=\frac{W_{wet}-W_{dried}}{W_{dried}}\times 100$$

#### 3.5.6. Enzymatic Degradation

The degradation of the 3D-printed collagen-based gels with and without Ag was evaluated by collagenase digestion. Briefly, the Col and Col-Ag gels were first weighed and placed in a 24-well plate. Then, 500 μL of a 50 U/mL solution of type I collagenase enzyme (Gibco^®^, 340 U/mg) was added to each of the wells containing the 3D-printed scaffolds and incubated at 37 °C. The weight of the Col and Col-Ag gels was measured after 0.5 h, 1 h, 2 h, 3 h, 4 h, 5 h, and 24 h. For this, the material was removed from the enzyme solution, the excess of liquid was dried and finally weighted. The changes of weight of Col and Col-Ag gels were analyzed over time.

#### 3.5.7. Silver Content in 3D-Printed Collagen-Based Gels

To determine the final concentration of silver in 3D-printed Col-Ag gels, Col-Ag0.05-2, Col-Ag0.05-4, Col-Ag0.05-6, Col-Ag0.1-2, Col-Ag0.1-4, and Col-Ag0.1-6 were digested with 5 mL of an acid mixture of HNO_3_ (65%)/H_2_O_2_ (10 vol.) 9:1 for 24 h, when a complete degradation of collagen-based gels was reached. The final silver concentration was determined by Atomic Absorption Spectrometry in a 210 VGP spectrometer (Buck Scientific, Norwalk, CA, USA) by the electrothermal atomization method using pyrolytic graphite tubes.

#### 3.5.8. Silver Release

To study the release of Ag+ from the 3D-printed collagen scaffolds containing in situ prepared AgNPs, the materials were submerged in 500 μL of H_2_O and incubated at room temperature for 24 h. The concentration of silver in the supernatant was measured by Atomic Absorption Spectrometry in a 210 VGP spectrometer (Buck Scientific) by the electrothermal atomization method using pyrolytic graphite tubes.

### 3.6. Antimicrobial Activity

To study antimicrobial activity of silver in 3D-printed Col-Ag gels, Col-Ag0.05-2, Col-Ag0.05-4, Col-Ag0.05-6, Col-Ag0.1-2, Col-Ag0.1-4, and Col-Ag0.1-6 Gram-positive (*Staphylococcus aureus*, ATCC 29213) and Gram-negative (*Escherichia coli*, ATCC 9637) bacteria were used. The antimicrobial activity was evaluated using two methods: the solid and the dilution method. Both, Gram-negative and positive bacteria were grown in Luria Bertani (LB) medium (yeast extract, 5 g/L; NaCl, 10 g/L, and tryptone, 10 g/L) at 37 °C and diluted up to 1 × 10^6^ Colony Forming Units (CFU)/mL. Briefly, for the disk diffusion method, we spread 50 µL of a bacterial suspension 1:1000 on an agar Petri dish. Then, the 3D-printed Col-Ag gels, Col-Ag0.05-2, Col-Ag0.05-4, Col-Ag0.05-6, Col-Ag0.1-2, Col-Ag0.1-4, and Col-Ag0.1-6 were placed on the agar surface and incubated them at 37 °C. Finally, the diameter of the bacteria-free areas that surround the 3D-printed Col-Ag materials to determine the antimicrobial activity was measure. In addition, the mentioned groups were compared to the antimicrobial activity of Ag (Nanotek S.A., 20–40 nm, colloidal suspension of 1% *w/v*, (nanArgen^®^, Buenos Aires, Argentina), CAS no. 7440–22-4) in different concentrations. On the other hand, for the dilution method, 100 µL of the bacterial suspension 1:1000 was mixed with 3D-printed Col-Ag gels, Col-Ag0.05-2, Col-Ag0.05-4, Col-Ag0.05-6, Col-Ag0.1-2, Col-Ag0.1-4, and Col-Ag0.1-6 with 900 µL of LB medium, and were incubated at 37 °C for 24 h. Then, 20 µL of the dilutions was seeded on agar Petri dishes and incubated at 37 °C for 24 h. Finally, CFU was determined by counting manually the colonies on the plate. To determine the minimum inhibitory concentration (MIC) of the material, Col-Ag0.05-6 was selected, since it is the condition with fewer concentration of Ag. Different amounts of the material were placed in the LB medium with *S. aureus* or *E. coli* and incubated for 24 h at 37 °C. Finally, the MIC was determined as the lowest concentration of the material at which there was no visible growth of the organism. The growth was evaluated by turbidimetry. Results are expressed as mean ± SD from triplicate experiments.

## 4. Conclusions

In this work, antimicrobial 3D-printed collagen-based scaffolds through in situ synthesizing AgNPs by the UV irradiation method were developed. First, lyophilized collagen was resuspended in AgNO_3_ (0.05 and 0.1 M) to prepare suitable inks for 3D printing. Once the scaffolds were 3D-printed, they were irradiated during different UV intervals (2, 4, 6 h). As a result, AgNPs with different morphologies and sizes were obtained within the collagen scaffolds. This is very interesting since it was possible to grow and control the shape and size of the Ag nanoparticles through an environmentally friendly and simple method, without using toxic reagents. DSC analysis showed that the concentration of AgNO_3_ and UV time exposure influence the thermal stability of collagen. This was also verified by the enzymatic degradation assay, which showed that 3D-printed Col-Ag scaffolds prepared from a higher concentration of AgNO_3_ were more resistant to collagenase action, indicating that a higher amount of silver particles acts as better reinforcement of the 3D-printed material. In addition to this, swelling capacity of Col-Ag also varied according to the preparation conditions of the antimicrobial 3D-printed materials. Finally, each and every Col-Ag scaffold showed bactericidal activity against Gram-positive and Gram-negative bacteria.

In summary, in this work the UV irradiation method and 3D printing technique were combined to in situ prepare AgNPs, and thus develop broad spectrum antimicrobial collagen-based scaffolds. This is attractive to design and prepare new bactericidal materials by nontoxic and cost-effective methodologies.

## Figures and Tables

**Table 1:** Size of AgNPs formed in situ, inside the 3D-printed collagen-based scaffolds.

Sample	AgNPs Size (nm)
Col-Ag0.05-2	26.17 ± 5.53
Col-Ag0.05-4	27.70 ± 7.45
Col-Ag0.05-6	24.39 ± 7.50
Col-Ag0.1-2	12.02 ± 3.34
Col-Ag0.1-4	9.74 ± 2.23
Col-Ag0.1-6	10.18 ± 2.45

## Data Availability

Not applicable.
